# Characterization and Function Analysis of the Beta-Carotene Oxygenase-like Genes in Carotenoids Metabolism of the Ridgetail White Prawn *Exopalaemon carinicauda*

**DOI:** 10.3389/fphys.2020.00745

**Published:** 2020-07-09

**Authors:** Yue Jin, Yang Yu, Chengsong Zhang, Shihao Li, Xiaojun Zhang, Fuhua Li

**Affiliations:** ^1^Key Laboratory of Experimental Marine Biology, Institute of Oceanology, Chinese Academy of Sciences, Qingdao, China; ^2^Laboratory for Marine Biology and Biotechnology, Qingdao National Laboratory for Marine Science and Technology, Qingdao, China; ^3^University of Chinese Academy of Sciences, Beijing, China; ^4^Center for Ocean Mega-Science, Chinese Academy of Sciences, Qingdao, China; ^5^The Innovation of Seed Design, Chinese Academy of Sciences, Wuhan, China

**Keywords:** carotenoid, oxygenases, beta-carotene oxygenase-like genes, carotene metabolism, *Exopalaemon carinicauda*

## Abstract

Carotenoids are almost universally distributed in living organisms. The oxidative metabolism by carotene oxygenase contributes to the metabolic processes of carotenoids. 15,15ʹ-beta-carotene oxygenase (BCO1) and 9ʹ,10ʹ-beta-carotene oxygenase (BCO2) are two important carotenoid oxygenases. In order to understand the function of carotenoid oxygenases in crustaceans, seven genes encoding carotenoid oxygenases (named *EcBCO-like*) were isolated from the transcriptome database of *Exopalaemon carinicauda*. After phylogenetic analysis with carotenoid oxygenases reported in other species, *EcBCO-like1*, *EcBCO-like3*, and *EcBCO-like6* were chosen for further functional study. The prawns after *EcBCO-like1* knockdown suffered continuous death, which suggested its important role for the survival of the animals. For the prawns after *EcBCO-like3* knockdown, no phenotype change was observed. The prawns after *EcBCO-like6* knockdown showed color changes in their hepatopancreas when they were fed with carotenoids-containing diet, and the content of carotenoid in their hepatopancreas was much higher than that in the control prawns. The present study will pave the way for further understanding the carotenoids metabolism in the prawns.

## Introduction

Carotenoids, a class of isoprenoid lipids, play an important role both in plants and animals. The carotenoids and their derivatives are colorants for feather or skin pigmentation as well as a type of antioxidant to protect eyesight and enhance immunity (Bone et al., 2000; Krinsky et al., 2003; Mcgraw and Ardia, 2005). Some carotenoids are the major dietary source for provitamin A, mainly beta-carotene (Sommer and Vyas, 2012). In crustaceans, carotenoids have also been reported with other functions. Feeding of carotenoids can improve the crustacean aquaculture and increase offspring survival rate (Yamada et al., 1990). Carotenoids and retinoids are also important for reproduction and growth (Dall, 1995; Liñán-Cabello et al., 2004; Liñán-Cabello and Paniagua-Michel, 2004). For animals, who are unable to *de novo* synthesize carotenoids, the carotenoids are usually acquired from diets. Similarly, vitamin A cannot be synthesized *de novo* in animals, it is produced either by oxidative cleavage of beta-carotene or other dietary provitamin A carotenoids (von Lintig and Vogt, 2004).

The metabolic process of carotenoid in animals includes a series of metabolic reactions. Degradation of carotenoids by enzymes is the key step for the utilization of carotenoids. Till present, two types of carotene oxygenases are identified in vertebrates, which are annotated as 15,15′-beta-carotene oxygenase (BCO1) and 9′,10′-beta-carotene oxygenase (BCO2) (Wyss et al., 2000; Kiefer et al., 2001). BCO1 can produce two molecules of all-trans-retinal from carotenoids *via* the symmetric cleavage of beta-carotene at 15,15′ double bond, which is thought to be the main process for producing retinoid from carotenoids (Hessel et al., 2007; Amengual et al., 2013). Literatures suggested that the bioconversion roles of carotenoid metabolites into retinoids occurred in prawns (Liñán-Cabello et al., 2002). Beta-carotene can be converted to retinol in a process catalyzed by carotenoid oxygenase. Additionally, it was also proposed that oxygenic carotenoids such as canthaxanthin and astaxanthin might also be cleaved in crustacean, while only one molecule of retinoids was produced per molecule of carotenoids, respectively. In general, the role of carotenoids as precursors of retinoids in crustaceans was still less studied. Relative to BCO1, BCO2 can asymmetrically cleave beta-carotene, generating beta-10′-apocarotenal and beta-ionone, and beta-10′-apocarotenal can be cleaved by BCO1 to produce retinaldehyde and downstream derivatives including retinol and retinoic acid (Kiefer et al., 2001; Amengual et al., 2013; Dela Sena et al., 2013).

It was reported that knockout of BCO1 could lead to vitamin A deficiency, while supplement of beta-carotene in diet caused an accumulation of beta-carotene in the liver of animals (Hessel et al., 2007). BCO2 has been considered as an alternative source of vitamin A production (Eroglu and Harrison, 2013; Wu et al., 2016). Additionally, BCO2 can also catalyze oxidative cleavage of xanthophylls like zeaxanthin and lutein to generate rosafluene and ionones (Kiefer et al., 2001; Dela Sena et al., 2016). The two BCOs share 40% sequence homology and are both expressed in tissues such as liver, intestine, and kidney (Raghuvanshi et al., 2015). Actually, animal BCO are members of the carotenoid cleavage oxygenase (CCO) superfamily, which also includes other members, such as carotenoid isomerooxygenase (NinaB) in *Drosophila* (Oberhauser et al., 2008), carotenoid cleaving enzymes in plants (CCDs) and so on (Poliakov et al., 2017).

Currently, carotenoid oxygenases have been reported in various species, including *Drosophila*, chicken, mice, and human (von Lintig et al., 2005; von Lintig, 2010). It was proved that BCO1 expression was necessary for production of retinal from provitamin A carotenoids (Hessel et al., 2007; van Helden et al., 2010). BCO2 was regarded as having a function in preventing excessive accumulation of carotenoids in mitochondria (Lobo et al., 2012). A SNP mutation in BCO2 gene caused an accumulation of lutein in tissues, resulting in a yellow fat phenotype in sheep (Våge and Boman, 2010). Mutations inhibited the expression of BCO2 enzyme in the yellow skin chicken and resulted in the deposition of uncleaved carotenoids (Eriksson et al., 2008). Genetic disruption of BCO2 can also increase the concentration of dietary pigment in plasma and blood of the mice (Babino et al., 2015).

Although many studies have demonstrated that carotenoid oxygenases played critical roles in the formation and metabolism carotenoid metabolites, the function of carotenoid oxygenases in prawns is not clear. In the ridgetail white prawn (*Exopalaemon carinicauda*), five types of carotenoid pigment were identified, including astaxanthin, unidentified carotenoid, canthaxanthin, echinenone and beta-carotene (Zhang et al., 2018); however, limited research about the carotenoid metabolism is reported in prawn. In this study, genes encoding carotenoid oxygenases were identified in the ridgetail white prawn *E. carinicauda*, and their roles in carotenoid metabolism were investigated. These results will be useful for revealing the metabolism of carotenoids in crustaceans.

## Materials and Methods

### Animals for Gene Expression Analysis

A total of 10 ridgetail white prawns were collected from the indoor tanks in the laboratory from Institute of Oceanology, Chinese Academy of Sciences, Qingdao, China. The average body length was 4.38 ± 0.42 cm and the average body weight was 0.98 ± 0.31 g. Different tissues, including heart, hepatopancreas, gill, muscle, stomach, intestine, hemocytes, epidermis, eyestalk, ventral nerve cord, and thoracic ganglia were collected from these individuals. Hemolymph was first collected using a sterile syringe preloaded with equal volume of anticoagulant (115 mmol L^−1^ glucose, 27 mmol L^−1^ sodium citrate, 336 mmol L^−1^ NaCl, and 9 mmol L^−1^ EDTA·Na_2_·2H2O, pH 7.4), then hemocytes were immediately harvested by centrifugation at 800 *g*, 4°C, for 10 min. These tissues were immediately frozen in liquid nitrogen for total RNA extraction and tissue expression test.

### Total RNA Extraction and Synthesis of cDNA

The total RNA was extracted separately from different tissues by RNAiso Plus reagent (TaKara, Japan) according to the manufacturer’s instructions. The quality of extracted RNA was assessed by electrophoresis on 1% agarose gel and quantified by NanoDrop 2000 spectrophotometer (Thermo Fisher Scientific, USA). About 1 μg of the total RNA was used for complementary DNA (cDNA) generation with PrimeScript RT reagent Kit (TaKaRa, Japan). According to the manufacturer’s instructions, gDNA Eraser was firstly used to remove the genomic DNA (gDNA), and then the first strand cDNA was synthesized by PrimeScript RT Enzyme with random primers.

### Characterization of EcBCO-like Genes and Phylogenetic Analysis

The corresponding genes were obtained by blasting the BCO genes with the assembled transcriptome database of the ridgetail prawn E. carinicauda. Additionally, PCR and Sanger sequencing were used to verify those sequences of EcBCO-like genes. The complete open reading frame (ORF) region and amino acid sequence of EcBCO-like genes were deduced using ORF finder^1^. Conserved protein domains were predicted with InterPro^2^ (Finn et al., 2017) and SMART^3^ (Letunic et al., 2015) databases. The amino acid sequences of BCOs from different species for phylogenetic analysis were obtained from the NCBI database (NCBI^4^). Then the phylogenetic analysis was performed by MEGA7.0 software (Kumar et al., 2016), and the phylogenic tree was construct by the Neighbor-Joining distance algorithm (Zhang and Sun, 2008). The bootstrapping test was adopted with 1,000 replications and the evolutionary distances were computed using the p-distance method (Nei and Kumar, 2000).

### Quantitative Real-Time PCR

The quantitative real-time PCR (qPCR) analysis was performed to detect the relative expression level of EcBCO-like genes in different tissues using the Eppendorf Mastercycler ep realplex (Eppendorf, Germany). The 18S rRNA gene was used as an internal reference. Amplification procedures were as following: denaturation at 94°C for 2 min, followed by 40 cycles of 15 s at 95°C, 15 s at 56°C or 57°C, and 20 s at 72°C, and a final 20-min melting curve step was added to test the specificity of these primers. All samples for qPCR were performed in quadruplicates. Then the relative gene expression levels were calculated by following the comparative Ct method with the analysis formula 2^−ΔΔCt^ (Livak and Schmittgen, 2000; Johnson et al., 2014). The information of related primers is listed in Table 1.

**Table 1:** Information of primers used for quantitative real-time PCR (qPCR).

Amplified unigenes	Sequence (5ʹ to 3ʹ)	Tm (°C)
*EcBCO-like1*	qF: GCGGACCTTGATAGCAGATG	56
	qR: TACCCCAACCCTGAAATCCT	
*EcBCO-like2*	qF: CTTCCTCGTAGGCGTTGGTGT	57
	qR: CAGCCGTTGGTATTTAGGTTTG	
*EcBCO-like3*	qF: GCAGAGGTTTATGAGTGCGTTTA	56
	qR: ACCTTGTCTTTTCGCCTACGG	
*EcBCO-like4*	qF: TCGTAGCGACAACAGAAGCT	57
	qR: GGGCAATTCTTTCACCGGAG	
*EcBCO-like5*	qF: GGTGATGACGATTCGGTTGG	57
	qR: GATGGAAGGACGAGTCATAAGG	
*EcBCO-like6*	qF: CCTACAAGCGACAAGACCACA	56
	qR: TTCAGCAGGCTGCAAAACTC	
*EcBCO-like7*	qF: CTGATACTGTGCGAGTGGTGG	56
	qR: TCCTGCTTTGTGGGAGATGAT	
*18S rRNA*	qF: TATACGCTAGTGGAGCTGGAA	55
	qR: GGGGAGGTAGTGACGAAAAAT	

### dsRNA Synthesis and Dosage Optimization of dsRNA Injection

Based on the tissue expression and the annotation of the EcBCO-like genes in the prawn, we selected three genes including *EcBCO-like1*, *EcBCO-like3*, and *EcBCO-like6* for further functional analysis using RNA interference (RNAi) method. The cDNA synthesized above was used as template to amplify the three EcBCO-like genes. The related primers for amplifying the three EcBCO-like genes are shown in Table 2. The PCR products were purified using MiniBEST DNA Fragment Purification Kit Ver.4.0 (Takara, Japan), then ligated with vector pMD-19 T (Takara, Japan), and transformed into TRans5α *Escherichia coli*. The clone was selected and sequenced to confirm the accuracy of the target sequence. Then the plasmid was extracted using Plasmid DNA Mini Kit (OMEGA, USA) according to the manufacturer’s protocol. For double-stranded RNA (dsRNA) synthesis, *EcBCO-like1*, *EcBCO-like3*, and *EcBCO-like6* were amplified with the primers containing T7 promoter (Table 2), and the recombinant plasmids were used as templates. Primers of *dsEGFP* with the T7 promoter sequences were also used to clone a 289 bp DNA fragment of enhanced green fluorescent protein (EGFP) gene based on pEGFP-N1 plasmid (Clontech, Japan). Then the PCR products were assessed by electrophoresis on 1% agarose gel and purified using MiniBEST DNA Fragment Purification Kit Ver.4.0 (TaKaRa, Japan). The purified products were used to synthesize the corresponding dsRNAs using TranscriptAid T7 High Yield Transcription Kit (Thermo Fisher Scientific, USA). The method for synthesis and purification of dsRNA was the same as described previously (Wang et al., 2016). Redundant single-strand RNA was digested by RNaseA (TaKaRa, Japan). Finally, the quality of synthesized dsRNA was assessed by electrophoresis on 1% agarose gel and quantified by NanoDrop 2000 spectrophotometer (Thermo Fisher Scientific, USA).

**Table 2:** Information of primers used for dsRNA synthesis.

Name of genes	Sequence (5ʹ to 3ʹ)
*EcBCO-like1*	F: ACCATCGTCCTCATCTATTGCAT
	R: ATAAGCCCTTCCTAGTCTTCCAC
*EcBCO-like3*	F: TACAGGAGAACTCATCAAGACGG
	R: GTTCACTGACTAGCTTCTCTCCA
*EcBCO-like6*	F: CTCGTTTGGTTGAGGCAGTG
	R: CCTTGTCTTTTCGCCTACGG
*dsEcBCO-like1*	F: TAATACGACTCACTATAGGGACCATCGTCCTCATCTATTGCAT
	R: TAATACGACTCACTATAGGGATAAGCCCTTCCTAGTCTTCCAC
*dsEcBCO-like3*	F: TAATACGACTCACTATAGGGTACAGGAGAACTCATCAAGACGG
	R: TAATACGACTCACTATAGGGGTTCACTGACTAGCTTCTCTCCA
*dsEcBCO-like6*	F: TAATACGACTCACTATAGGGCTCGTTTGGTTGAGGCAGTG
	R: TAATACGACTCACTATAGGGCCTTGTCTTTTCGCCTACGG
*dsEGFP*	F: TAATACGACTCACTATAGGGCAGTGCTTCAGCCGCTACCC
	R: TAATACGACTCACTATAGGGAGTTCACCTTGATGCCGTTCTT

The underlined part is T7 promoter sequence.

In order to obtain the optimal dose for RNAi, pre-experimental exploration for each BCO-like gene was conducted. A total of 65 individuals with an average body length of 5.20 ± 0.40 cm and body weight of 1.43 ± 0.27 g were randomly separated into five groups including dsEcBCO-like1, dsEcBCO-like3, dsEcBCO-like6, dsEGFP, and phosphate buffered saline (PBS) group. Each dsRNA injected group contained 15 prawns and those prawns were then divided into three sub-groups with five individuals. In the groups for dsRNA injection, different doses including 2, 4, and 6 μg dsRNA were set for each sub-group. The group PBS included five prawns and they were injected with the equal volume of PBS. At last, dsRNA and PBS were injected into the abdominal segment of each prawn separately. At 48 h after injection, the total RNA from cephalothoraxes in group PBS and each sub-group was extracted and the corresponding gene expression was detected by qPCR according to section qPCR to analyze the RNAi efficiency. After dosage optimization, we conducted a second pre-experiment to explore the duration of interference effect with the optimal dosage. The interference efficiency on days 2, 6, and 10 was evaluated to determine the injection interval for long time interference experiment.

### RNA Interference Experiment and Carotenoid Feeding Experiment

In the formal experiment, another 180 individuals with an average body length of 5.25 cm and an average body weight of 1.50 g were cultured in tanks with aerated seawater at 24°C. They were divided into six groups with 30 individuals in each group, including one PBS, dsEcBCO-like1, dsEcBCO-like3, dsEcBCO-like6, and two dsEGFP groups (one is the same as the concentration of *dsEcBCO-like1* and the other is the same as the *dsEcBCO-like3* and *dsEcBCO-like6*). Each group contained three replicates, with 10 prawns in each. Prawns in three RNAi groups were injected with optimal amount of dsRNA for each EcBCO-like gene. In addition, same amount of dsRNA of EGFP and the same volume of PBS were set as negative controls. These prawns were used for carotenoid feeding experiment described in the following section. During the feeding experiment, those dsRNA or PBS were injected again after 1 week. The experiment lasted for 2 weeks. At the last of the experiment, the effect of interference was checked by qPCR according to section qPCR.

Beta-carotene was purchased from LTD Xinchang Pharmaceutical Factory (Zhejiang, China), and zeaxanthin and lutein were from SPRING-BIO Company (Zhejiang, China). These carotenoids (zeaxanthin, lutein, and beta-carotene) used in the feeding experiment were delivered into the prawns by incorporating into the diet (100 mg kg^−1^ for each). Both the RNAi group and the control group (EGFP and PBS) were fed with the same prepared diet. These prawns were fed 3–4 times a day. In addition, another control group including 30 prawns (10 prawns in each replicate) was set, and they were cultured together with the 180 prawns as described above. These 30 prawns were with an average body length of 5.08 cm and an average body weight of 1.47 g, and they were not injected with any dsRNA or PBS. Prawns in this control group were fed with the base diet without any pigment. The feeding experiment lasted for 14 days. At the end of the experiment, the muscle, hepatopancreas and eyestalk of these prawns were collected and immediately frozen in liquid nitrogen, and stored at −80°C until use.

### Carotenoid Extraction and High-Performance Liquid Chromatography Analysis

The carotenoid standards for high-performance liquid chromatography (HPLC) analysis, including lutein, zeaxanthin, and beta-carotene, were purchased from Sigma-Aldrich (St. Louis, MO, USA). Methanol, acetonitrile, and acetyl acetate (Thermo Fisher Scientific, USA) used for pigment separation are HPLC-grade solvent.

All the tissues were freeze-dried in a freeze-dryer (SCIENTZ-10N, Ningbo Scientz Biotechnology CO., China) and ground into powder. Carotenoids were extracted from tissues (20–30 mg) under the dim safety light. The carotenoids in those tissues were extracted through grinding using a solvent mixture of dichloromethane/methanol (25:75 v/v) (Boussiba et al., 1999). After centrifugation at 10,000 *g*, 4°C, for 3 min, organic layer was transferred into another new tube. The above extraction steps were repeated until the sample was totally white. At last, all organic layers were pooled together and centrifuged at 10,000 *g* for 10 min. Then the supernatant was passed through a 0.22 μm syringe filter into HPLC vials and analyzed by HPLC using carotenoids standard.

For the HPLC analysis, an Agilent 1200 HPLC equipped with an Rx-C18 analytical column (4.66250 mm) and a Quatpump (Agilent Technologies Inc., SantaClara, CA, USA) were used. The specific method was the same as previously described (Xie et al., 2013). The injection volume of each sample was 20 μl, and the flow rate was 0.8 ml min^−1^. Briefly, the initial solvent, consisting of water:methanol:acetonitrile:acetyl acetate (5:30:65:0), was used to balance the column for 10 min. The pigments’ separation was started by a 5-min linear gradient from the initial solvent to water:methanol:acetonitrile:acetyl acetate (0:15:85:0), and then isocratically continued for 7 min. Eluents were then transited to water: methanol: acetonitrile: acetyl acetate (0:45:35:20) within 2 min, followed by 16-min linear gradient to water: methanol: acetonitrile: acetyl acetate (0:45:0:55). Absorbance was measured at 450 nm as indication.

The content of beta-carotene was quantified based on the sample weight, peak area and standard curve of beta-carotene content. We took the peak area into the standard curve to calculate the beta-carotene content of the sample. The calculated value was set as C. Other conditions that might affect the quantification, such as sample volume, injection volume, unit conversion, and so on, were set exactly the same in these groups. Then the formula can be simplified as X = C/M, where the letter X represents the content of beta-carotene and the letter M represents the sample weight used for carotenoid extraction.

### Statistical Analyses

The statistical significances between controls and different treatments were subjected to one-way ANOVA by using SPSS (version 20). The significant difference at *p* < 0.01 was labeled with double asterisks.

## Results

### Diversity of BCO-like Genes in *E. carinicauda*

**Figure 1:**
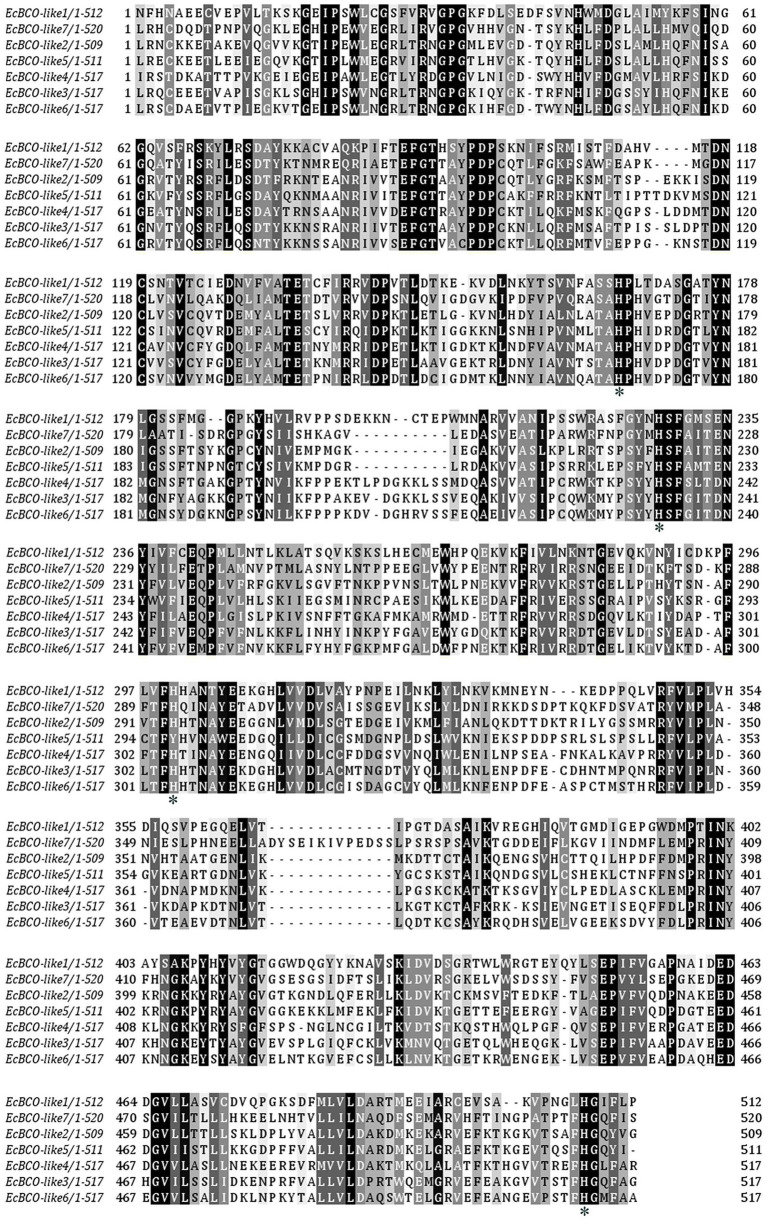
Alignment of conserved domain sequences in EcBCO-like genes. Black color represents the identical amino acids (aa) among sequences. The four conserved histidine residues related to Fe^2+^ binding site are marked with asterisks (*).

According to the sequences of carotenoid oxygenases reported in NCBI, nine homologous genes were identified in the transcriptome database of ridgetail white prawn. Seven of them (GenBank accession numbers from MN906761 to MN906767) were annotated as BCO, which were designated as EcBCO-like1–EcBCO-like7. The ORFs of EcBCO-like genes were 1566, 1584, 1617, 1713, 1596, 2082, and 1683 base pairs (bp), encoding 195, 527, 538, 570, 531, 693 and 560 amino acids (aa), respectively. SMART domain prediction and NCBI online domain analysis of those amino acid sequences showed that they all contained RPE65 conserved domain. The domain of EcBCO-like genes and their deduced amino acid sequences are shown in Figure 1. The four conserved histidine residues related to Fe^2+^ binding site were also found in the deduced EcBCO-like protein. The other two sequences were annotated as carotenoid isomerooxygenase-like, which were named as *carotenoid isomerooxygenase-like1* and *carotenoid isomerooxygenase-like2*.

### Phylogenetic Analysis of EcBCO-like Genes

**Figure 2:**
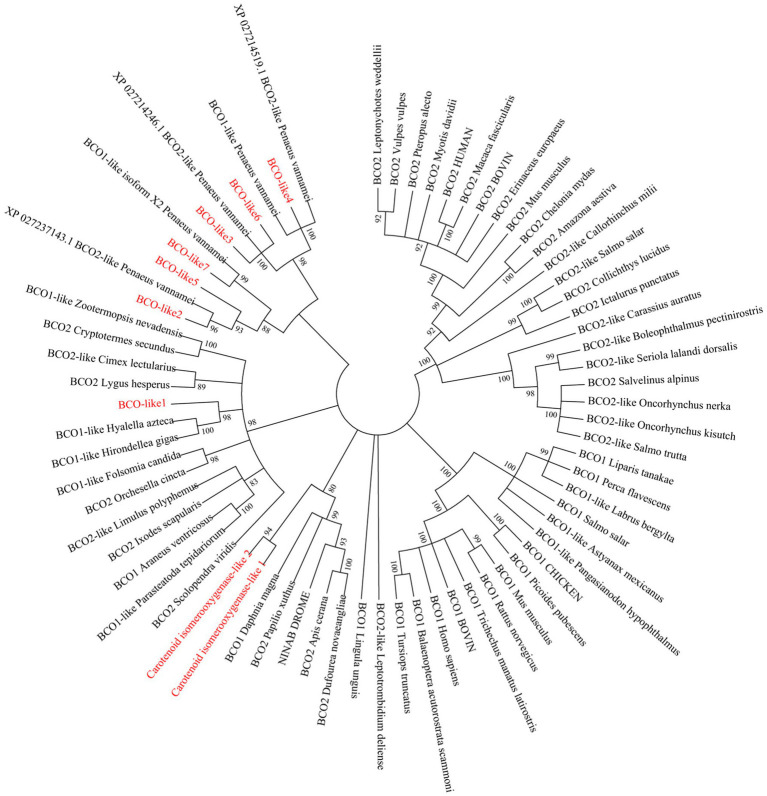
Evolutionary relationships of carotenoid oxygenases from *Exopalaemon carinicauda* and other species. The phylogenetic tree was constructed by the Neighbor-Joining distance algorithm, and bootstrap value was set at 1,000. All the EcBCO-like genes and two carotenoid isomerooxygenase like genes in the tree are marked with red font.

Phylogenetic analysis showed that the BCO1 and BCO2 in vertebrates were separately clustered. For the invertebrate species, the reported BCO1-like genes and BCO2-like genes were not clustered (Figure 2). The EcBCO-like genes in prawns completely separated from carotenoid oxygenases in vertebrates, indicating that EcBCO-like genes were different from the BCO1 and BCO2 in vertebrates. Among these EcBCO-like genes, EcBCO-like1 was grouped together with BCO1-like in *Hyalella azteca* and *Hirondellea gigas*, which was the distant cluster with the vertebrate carotenoid oxygenase genes. The EcBCO-like2, EcBCO-like5, and EcBCO-like7 showed a closer evolutional relationship. The EcBCO-like3, EcBCO-like4, and EcBCO-like6 were clustered together. Additionally, the two carotenoid isomerooxygenase-like genes in the prawn were distant with the seven EcBCO-like genes. From the view of the phylogenetic analysis, the carotenoid oxygenase genes in *E. carinicauda* were similar with the corresponding genes in *Penaeus vannamei* (*L. vannamei*) and they were clustered together.

### Expression of EcBCO-like Genes in Different Tissues

**Figure 3:**
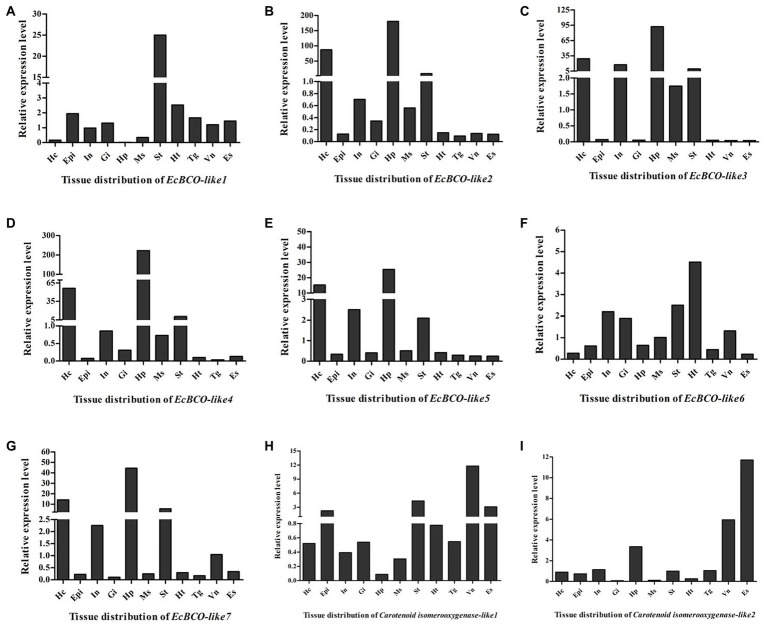
Tissue distribution of *EcBCO-like1* **(A)**; *EcBCO-like2* **(B)**; *EcBCO-like3* **(C)**; *EcBCO-like4* **(D)**; *EcBCO-like5* **(E)**; *EcBCO-like6* **(F)**; *EcBCO-like7* **(G)**; *carotenoid isomerooxygenase like1* **(H)** and *carotenoid isomerooxygenase like2* **(I)**. Hc, hemocytes; Epi, epidermis; In, intestine; Gi, gill; Hp, hepatopancreas; Ms., muscle; St, Stomach; Ht, heart; Tg, thoracic ganglia; Vn, ventral nerve cord; and Es, eyestalk.

Tissue distribution analysis of EcBCO-like genes showed that different genes displayed different expression profiles (Figure 3). From the phylogenetic analysis result, the *EcBCO-like1* and *EcBCO-like7* were similar to the BCO1 family; however, *EcBCO-like1* was highly expressed in stomach while *EcBCO-like7* was highly expressed in hepatopancreas and hemocytes. The other genes were similar to BCO2-like genes. *EcBCO-like6* was highly expressed in heart, stomach, intestine and gill while all the other BCO2-like genes were highly expressed in hepatopancreas, hemocytes, stomach, and intestine. Compared with the seven EcBCO-like genes, *carotenoid isomerooxygenase-like1* and *carotenoid isomerooxygenase-like2* were highly expressed in the ventral nerve cord and eyestalk.

Combined with phylogenetic and tissue expression analysis, three representative genes were selected for the further function analysis. The EcBCO-like1 gene was selected because it was distant with the other EcBCO-like genes based on the phylogenetic result. EcBCO-like genes, including *EcBCO-like2*, *EcBCO-like3*, *EcBCO-like4*, *EcBCO-like5*, and *EcBCO-like7*, were clustered into one group and all highly expressed in the hepatopancreas. *EcBCO-like3* was randomly selected as a representative gene from them for further study. Although *EcBCO-like6* was also clustered together with these genes, it showed different tissue distribution patterns from them. Therefore, *EcBCO-like6* was also selected for functional study.

### RNA Interference Efficiency of EcBCO-like Genes

**Figure 4:**
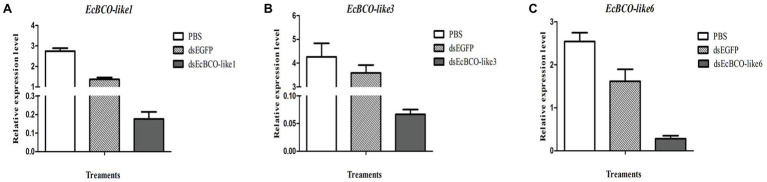
The mRNA expression of EcBCO-like genes after injection of dsRNA. **(A)** mRNA expression of *EcBCO-like1*. **(B)** mRNA expression of *EcBCO-like3*. **(C)** mRNA expression of *EcBCO-like6*. Two microgram double-stranded RNA (dsRNA) of *EcBCO-like1*, four microgram dsRNA of *EcBCO-like3* and *EcBCO-like6* was injected into one individual, respectively. Significant differences of the expression levels between treatment group (*EcBCO-like* dsRNA injection group) and control group (*EGFP* dsRNA and PBS injection group) were identified.

It was confirmed that 2 μg dsRNA was the effective dose for *EcBCO-like1*, but for *EcBCO-like3* and *EcBCO-like6*, 4 μg was more effective. Results showed that the interference efficiency began to diminish after 6 days. Considering the convenience of setting the duration of interference injection, the injection was conducted once a week. The gene expression was detected by qPCR. Results of qPCR analysis showed that the mRNA levels of *EcBCO-like1*, *EcBCO-like3*, and *EcBCO-like6* were about 90% downregulated compared with the dsEGFP and PBS injected groups (Figure 4).

### Effects of Knockdown of EcBCO-like Genes on the Content of Carotenoid

**Figure 5:**
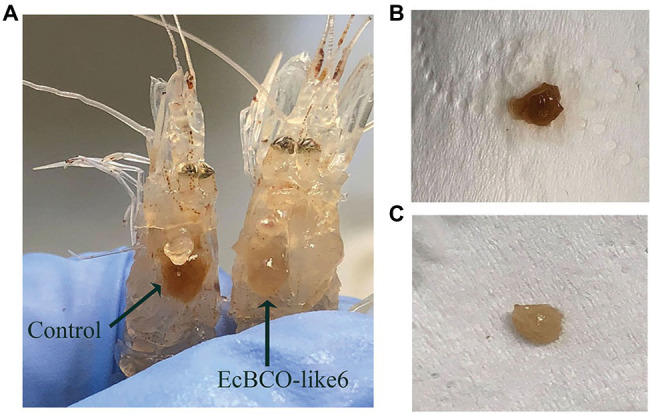
The color changes of hepatopancreas in prawns after *dsEcBCO-like6* injected. **(A)** represents the prawns in the control group and *dsEcBCO-like6* injected group; **(B)** shows the hepatopancreas of prawns in the control group feeding the same carotenoid; **(C)** represents the hepatopancreas of prawns in the EcBCO-like6 group.

During the experiment, the food-intake dropped significantly after 1 week of RNAi. For the dsEcBCO-like1 group, a high mortality rate was observed, and consequently, only seven prawns survived at the end of the experiment. For the dsEcBCO-like3 group, no appearance difference was observed between the RNAi group and the control group. For those prawns injected with dsRNA of *EcBCO-like6*, the hepatopancreas color was changed significantly compared to those of the control group (Figure 5).

**Figure 6:**
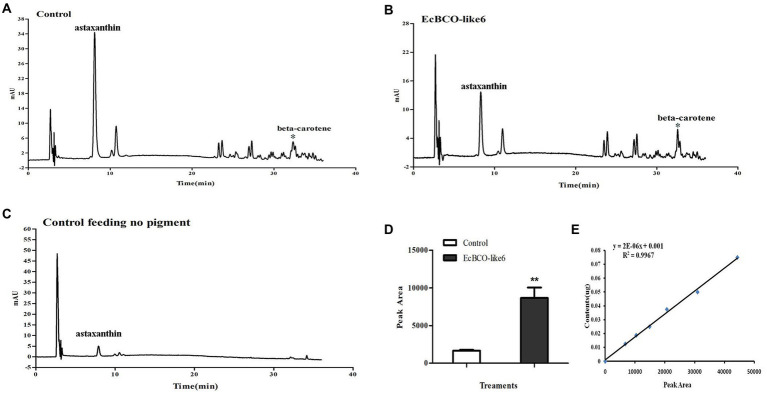
High-performance liquid chromatographies (HPLCs) and the standard curve of beta-carotene. **(A,B)** show the HPLC analysis of control group feeding the same pigment and the group *dsEcBCO-like6* injected, respectively; **(C)** shows the HPLC analysis of group without any pigment; **(D,E)** show the peak area and the standard curve of beta-carotene content. The main difference between the control group and EcBCO-like6 group was the peak area of beta-carotene. All samples were analyzed in three biological triplicates. Asterisks (**) indicate extremely significant differences (*p* < 0.01) of the peak area between EcBCO-like6 group and control group.

In view of the apparent phenotypic change of hepatopancreas in the EcBCO-like6 group, we detected their pigment content by HPLC analysis. The different peaks in the chromatograms contained astaxanthin, beta-carotene, and some unknown ones. The major peak in the control HPLC chromatograms (Figure 6, panel A) was astaxanthin. Beta-carotene was increased by five-times in the dsEcBCO-like6 group compared with that in the control group (Figure 6). Although some other peaks also changed in the RNAi experiment, they were not the pigments (zeaxanthin, lutein, and beta-carotene) that we focused on. We also detected the beta-carotene in eyestalk and muscle. However, the result revealed that the beta-carotene was detected primarily in hepatopancreas of RNAi prawn, but not in eyestalk or the muscle. In addition, by comparing the group feeding the normal diet and the group feeding diet with three pigments, we only detected the beta-carotene, whereas the zeaxanthin or lutein could not be detected in the group feeding diet with three pigments.

## Discussion

The widely investigated carotenoid oxygenases were BCO1 and BCO2 in vertebrates, of which BCO1 can catalyze the oxidative cleavage of provitamin A carotenoids *via* the symmetric cleavage of beta-caroteneat 15,15′ double bond, while BCO2 asymmetrically cleaves beta-carotene to yield an ionone and a beta-10′-apocarotenal. The function of carotenoid oxygenase genes in vertebrates were detailed investigated. However, in invertebrates, the function of carotenoid oxygenase genes was rarely studied. In *Drosophila*, only one kind of carotenoid oxygenases was found, which was encoded by the gene *ninaB* (von Lintig et al., 2001). In mollusk Yesso scallop (*Patinopecten yessoensis*), *PyBCO-like1*, was identified and it was proved that its down-regulation in muscle led to carotenoid deposition due to impaired carotenoid degradation (Li et al., 2019). According to the assembled transcriptome database of *E. carinicauda* and the sequences of carotenoid oxygenases reported in NCBI, several homologous genes were found. They were annotated as beta, beta-carotene 15,15′-oxygenase-like or beta, beta-carotene 9′,10′-oxygenase-like. Interestingly, these EcBCO-like genes except *EcBCO-like1* in prawns were clustered together and completely separated from other invertebrate carotenoid oxygenase genes. The EcBCO-like1 is more similar to the BCO1-like in *H. azteca* and *H. gigas*, indicating that they might have a common ancestor gene. The other EcBCO-like genes were also different from the BCO1 and BCO2 genes in vertebrates. These data raised our attention to investigating the function of BCO-like genes in *E. carinicauda*.

Previously, investigations were mainly focused on the substrate specificity, kinetics of beta-carotene cleavage, and cellular and subcellular expression of BCOs to better understand the metabolism of carotenoids (Raghuvanshi et al., 2015; Dela Sena et al., 2016). In the chicken, BCO1 is located in cytoplasm with a relatively high expression level in liver (Paik et al., 2001; Raghuvanshi et al., 2015). By contrast, BCO2 is located in the inner mitochondrial membrane (Palczewski et al., 2014). BCO genes also showed different tissue distribution patterns in different species. For example, BCO2 was highly expressed in the liver and testes of mice, whereas it was hardly detected in the liver of chickens. In our experiment, except *EcBCO-like6*, which was highly expressed in the heart, other EcBCO-like genes were highly expressed in hepatopancreas or stomach. This indicated that stomach and hepatopancreas might be major organs for digestion and metabolism of carotenoids. Notably, multiple BCO-like genes in the prawn might indicate their different roles in carotenoids metabolism. Additionally, both *carotenoid isomerooxygenase-like1* and *carotenoid isomerooxygenase-like2* were highly expressed in the eyestalk, indicating that they might have biological functions different from EcBCO-like genes. In *Drosophila melanogaster*, the *carotenoid isomerooxygenase*, which was encoded by *NinaB* gene, could convert carotenoids into retinoids (Oberhauser et al., 2008). The biological functions of them need to be further investigated.

On the basic knowledge about the evolutionary relationships and tissue expression of EcBCO-like genes from *E. carinicauda*, three genes including *EcBCO-like1*, *EcBCO-like3*, and *EcBCO-like6* were further analyzed to investigate their function in carotenoid metabolism. Among those identified seven genes, *EcBCO-like1* was selected as it did not group with the other EcBCO-like genes in the phylogenetic analysis. It was highly expressed in stomach, also showed a certain expression level in heart, epidermis, eyestalk, and ventral nerve cord. However, its expression level was low in hepatopancreas. After knockdown of *EcBCO-like1*, the prawns showed obvious decrease in food intake and the mortality rate increased rapidly. It indicated that this gene was crucial for survival of prawns. Prawns are unable to *de novo* synthesize carotenoids, which are usually acquired from diets. Carotenoids mainly participate in pigmentation, immune function, and antioxidation in crustaceans (Liñán-Cabello et al., 2002). Some studies also illustrate that carotenoids are important during reproduction and growth. In our experiment, knockdown of *EcBCO-like1* led to lethal of the prawns. Besides, the stage of the RNAi individuals was not good enough for the further function analysis, so little information was available for us to explain why the knock down of *EcBCO-like1* resulted in death. We inferred that knockdown of this gene altered the homeostasis and affected the metabolism of prawns. Similar result was reported in *Drosophila*. The mutations in BCO1 are associated with blindness of *Drosophila* (von Lintig et al., 2001). Overall, gene *EcBCO-like1* is likely to be critical to the physiological process of prawns.

Compared with prawns injected with *dsEcBCO-like1*, the prawns injected with *dsEcBCO-like6* did not suffer continuous death and were found to show color change in their hepatopancreas. This result suggested that the hepatopancreas might be severely affected after the gene was knocked down. We then detected the content of the pigment in hepatopancreas by HPLC and found that the beta-carotene in dsEcBCO-like6 group was significantly higher than that of the control animals. In fact, *EcBCO-like6* was only expressed at very low levels in the hepatopancreas. However, knockdown of *EcBCO-like6* caused color change of the hepatopancreas and variation in carotenoid levels. This might be because the hepatopancreas was the major organ for beta-carotene storage and metabolism. However, *EcBCO-like6* mainly participated in beta-carotene metabolism in other tissues. After knockdown of *EcBCO-like6*, the beta-carotene that was not metabolized in other tissues was transported to and accumulated in hepatopancreas. This led to the color change of the hepatopancreas and variation in carotenoid levels. In vertebrates, BCO2 plays an important role in the metabolism of nonprovitamin A carotenoids, such as lycopene, lutein, and zeaxanthin (Hu et al., 2006). It can cleave lutein to form the beta-ionone, beta-10′-carotenal, and apo-10,10′-carotenedialdehyde (Kiefer et al., 2001). However, lutein and zeaxanthin are not detected in hepatopancreas in our experiment, although they have been added to the diet. Therefore, we inferred that beta-carotene was more efficiently utilized by the prawn than zeaxanthin and lutein. The prawn might not absorb and utilize the zeaxanthin and lutein well.

In summary, our study identified seven EcBCO-like genes and two carotenoid isomerooxygenase-like genes in the ridgetail white prawns. These genes were characterized and three BCO-like genes were studied using RNAi method. Among them, two of which might cause metabolic changes. *EcBCO-like1* was crucial for the survival of prawns, and it might be of importance in fundamental metabolism process. *EcBCO-like6* played an important role in beta-carotene metabolism. The present results provide a preliminary understanding on the diversity and importance of BCO-like genes in the prawns, which need to be further investigated to reveal the functions of BCO-like genes in crustaceans.

## Data Availability Statement

The cDNA sequences of EcBCO-like genes in ridgetail white prawn *Exopalaemon carinicauda* can be found in the NCBI GenBank dataset with accession no. MN906761–MN906767 (https://www.ncbi.nlm.nih.gov/).

## Ethics Statement

This study was carried out in accordance with the recommendations of Welfare ethics of experimental animals and safety inspection system of animal experiments, laboratory animal management and ethics Committee of IOCAS. The protocol was approved by the laboratory animal management and ethics Committee of IOCAS.

## Author Contributions

YY and FL contributed conception and design of the study. YJ conducted the investigation, performed all experiments and wrote the manuscript. CZ, SL, and XZ collected data and performed the statistical analysis for the work. All authors contributed to the article and approved the submitted version.

### Conflict of Interest

The authors declare that the research was conducted in the absence of any commercial or financial relationships that could be construed as a potential conflict of interest.

## Footnotes

^1^https://www.ncbi.nlm.nih.gov/orffinder/

^2^http://www.ebi.ac.uk/interpro/

^3^http://smart.embl-heidelberg.de/

^4^http://www.ncbi.nlm.nih.gov/BLAST/
